# The White Paper and the New NHS

**Published:** 1989-11

**Authors:** 


### Editorial

### The White Paper and the New NHS

The debate on the Government White Paper goes on and, with an increasing general realisation of what is proposed, resistance is building up. From the hospitals point of view the nub of the proposals is the creation of an internal market in the NHS in which District Health Authorities purchase health care for their communities from wherever they think they can get the best bargains. This means that every service must be priced and evaluated, and contracts must be negotiated with the hospitals and other agencies providing them. In most instances both purchaser and provider are in a monopoly situation. Enormous cost and effort will go into the pricing and into the bargaining, it will be a field day for the suppliers and operators of computers, accountants, lawyers and administrators of all kinds. In the end Districts will go on placing patients in their local hospitals because that is where they want to go and with the present underprovision of services there is little choice anyway. This imaginary spur to efficiency is being introduced at a time when much real progress is being made with Management Budgeting Evaluation (1).

The internal market in the context of the British medical scene is an unreality. The deviser of the concept Professor Alain Enthoven is an American academic who was invited 5 years ago to take a look at our Health Services by the Secretary of the Nuffield Provincial Hospitals Trust and to give and after-dinner talk on it. He later expanded his ideas into an essay entitled 'Reflections on the Management of the NHS' and it is this that the government has latched on to. While pleased that his ideas are being considered he is nevertheless 'critical of the Governments insistence on rushing to implement the plans by 1991 without first having a trial, perhaps in a limited geographical area' (2). One guesses that his ideas may have formed originally with the American scene in mind where they may well be relevant. There rampant commercialism in medicine has resulted in costs of health provision reaching a level where even the wealthy have difficulty in paying health insurance premiums and some doctors are now unable to recoup their medical defence premiums from their earnings. In the US a state purchaser of services might bring some discipline into the health scene by introducing meaningful competition between all the independent providers. Professor Enthoven is reported to be worried that the Government has gone overboard on his ideas and to have said "I have no business telling people over here how they should deal with their problems when we in the US are in such a mess" (2). On the advice of a firm of American consultants a decade ago a health service reorganisation under Sir Keith Joseph created an entirely unneccessary Area Health Authority because it looked good on paper. Several years later it was quietly dismantled; the cost of the whole exercise in money and wasted effort can hardly be quantified. It looks as though history is repeating itself. A committee associated with the NHS Consultants Association headed by Professor Harry Keen of Guy's Hospital warns that "across the country much NHS money is being spent and many person hours diverted from medical care in preparation for and in some cases the implementation of the White Paper Directives before they have even been presented to Parliament let alone achieved the force of law". They believe that the Secretary of State is acting 'ultra vires' and propose to explore and if appropriate, to initiate a Judicial Review to question the lawfulness of his actions.

It unfortunately seems impossible for politicians, especially the present opportunist opposition, to restrain themselves from using the NHS for political advantage. The Labour party, ignoring the very real achievements of the NHS in provision of new services and in tightening financial discipline, knowing that expectation will always exceed realisation has remorselessly disparaged it. Mrs Thatcher, impatient for deliverance from the perpetual demand for more and more money has responded. This plan for a new NHS under Mr Kenneth Clarke could go even more badly wrong than the last one under Sir Keith Joseph. Unlike the Americans, our government does have a grip on expenses, it simply has to decide what the economy can afford, what services are worthwhile and to refuse to respond to parliamentary clamour and media pressure. It should take advice from its own professionals and spend its money providing more resources where they are short, and not abandon all caution and reality in a search for better bargains.

(1) Management Budgeting Evaluation Report. Radcliffe Infirmary, Oxford 1988.

(2) Prof Alain Enthoven interviewed by David Fletcher, Daily Telegraph, September 25th 1989.

